# Improving medical term embeddings using UMLS Metathesaurus

**DOI:** 10.1186/s12911-022-01850-5

**Published:** 2022-04-29

**Authors:** Ashis Kumar Chanda, Tian Bai, Ziyu Yang, Slobodan Vucetic

**Affiliations:** Department of Computer and Information Sciences, Temple University, Philadelphia, PA USA

**Keywords:** Electronic health records, EHR, UMLS, Medical terms, Embeddings, Natural language processing

## Abstract

**Background:**

Health providers create Electronic Health Records (EHRs) to describe the conditions and procedures used to treat their patients. Medical notes entered by medical staff in the form of free text are a particularly insightful component of EHRs. There is a great interest in applying machine learning tools on medical notes in numerous medical informatics applications. Learning vector representations, or embeddings, of terms in the notes, is an important pre-processing step in such applications. However, learning good embeddings is challenging because medical notes are rich in specialized terminology, and the number of available EHRs in practical applications is often very small.

**Methods:**

In this paper, we propose a novel algorithm to learn embeddings of medical terms from a limited set of medical notes. The algorithm, called *definition2vec*, exploits external information in the form of medical term definitions. It is an extension of a skip-gram algorithm that incorporates textual definitions of medical terms provided by the Unified Medical Language System (UMLS) Metathesaurus.

**Results:**

To evaluate the proposed approach, we used a publicly available Medical Information Mart for Intensive Care (MIMIC-III) EHR data set. We performed quantitative and qualitative experiments to measure the usefulness of the learned embeddings. The experimental results show that *definition2vec* keeps the semantically similar medical terms together in the embedding vector space even when they are rare or unobserved in the corpus. We also demonstrate that learned vector embeddings are helpful in downstream medical informatics applications.

**Conclusion:**

This paper shows that medical term definitions can be helpful when learning embeddings of rare or previously unseen medical terms from a small corpus of specialized documents such as medical notes.

## Background

Health providers use Electronic Health Records (EHRs) to keep information about their patient’s medical conditions and the procedures employed to treat them. While the primary purpose of EHRs is operational and administrative, EHRs have been increasingly useful in biomedical research studies such as patient phenotyping [1, 2], health risk prediction [3, 4], prediction of medical events [5, 6], medical code extraction [7], and relation extraction between medications and adverse drug effects [8]. Particularly, valuable parts of EHRs are medical notes, which are free text created by the medical staff to provide insights about the condition and treatment of patients. Extracting information and analysis of medical notes is an open machine learning (ML) problem. A critical pre-processing step in modern approaches for medical note analysis is medical term embedding, which refers to the representation of medical terms as vectors. Medical term embeddings can be used as inputs for neural networks in a range of predictive and descriptive tasks [9, 10]. In this paper, we refer to a medical term as a single word (e.g., *Parkinson*) or a multi-word (e.g., *Parkinson’s disease*) that is linked to an entry in a medical thesaurus, such as the UMLS Metathesaurus [11].

Recent research has resulted in several methods for learning embeddings of medical terms, diagnosis and procedure codes, medications, and lab tests [12–15]. In particular, the skip-gram model [16] is a popular choice for learning embeddings of terms both from general-purpose corpora (e.g., Wikipedia) and from specialized corpora (e.g., medical notes) [2, 13, 17] due to its simplicity and computational efficiency. The skip-gram and related embedding approaches, such as fastText [18], work well when a document corpus is large and when terms that need to be embedded are frequent. However, there are many applications that rely on relatively small corpora with an abundance of specialized terms and abbreviations [19–21], where direct application of the skip-gram model does not always result in high-quality embeddings.

The main contribution of this study is summarized as follows: we propose a new algorithm, called *definition2vec*, that is particularly appropriate for learning embeddings of infrequent or unobserved medical terms from a small corpus of medical notes. Our approach enhances the skip-gram algorithm by exploiting textual definitions of medical terms from existing publicly available resources, such as the UMLS Metathesaurus. We demonstrate experimentally that our algorithm provides useful embeddings of infrequent and unobserved medical terms and that those embeddings can increase the quality of downstream medical informatics tasks.

## Related work

Learning embeddings of n-grams, words, terms, sentences, and paragraphs is an active research topic due to the importance of embeddings in deep learning approaches for natural language processing. Modern embedding algorithms draw inspiration from the well-known distributional hypothesis, which states that words that occur in the same contexts tend to purport similar meanings [22]. An overview of traditional embedding approaches is provided in [23]. More recently, starting from seminal papers proposing skip-gram [16], GloVe [24], and fastText [18] algorithms, many general-purpose and specialized embedding algorithms were proposed both for processing text and various types of data objects such as sequences and graphs [25]. The skip-gram algorithm [16] learns embeddings as a by-product of predicting context words of a target word. FastText [18] is an alternative approach that treats words as sequences of n-grams that have their own embeddings and is sometimes useful in finding representations of out-of-vocabulary words.

Studying specialized approaches for embeddings of medical terms and concepts has been an active research area [2, 26–28]. The work on learning UMLS concept representations from medical notes and journals using the skip-gram algorithm [12, 13] is particularly relevant to this paper. A recent study [29] provides an extensive analysis of bio-medical word embeddings based on the skip-gram architecture. Med2Vec [17] is another relevant work that uses a two-layer neural network for learning embeddings of medical concepts from code occurrences and clinical narratives about patient visits. The authors of [30] proposed cui2vec that learns the embedding of UMLS Concept Unique Identifiers (CUIs) based on the distribution of concept co-occurrences in clinical notes. A related approach is described in [14] that focuses on temporal relations to embed medical concepts. It extends the Continuous Bag of Words (CBOW) model [16] to develop a time-aware attention approach for learning medical concepts. The research survey of Hahn et al. [31] provides a detailed overview of different medical information extraction methods that rely on medical term embeddings.

Other studies used external knowledge sources in different ways to improve embeddings and downstream predictive models [32, 33]. The authors in [32] combine UMLS Metathesaurus and Semantic Network information to learn concept embeddings following the Generative Adversarial Networks (GAN) framework [34]. Work in [33] uses the Medical Subject Heading (MeSH) term graph [35] to generate MeSH term sequences. While this previous work exploited known relations between medical terms, in our work, we leverage medical term definitions through an easy-to-implement and computationally efficient skip-gram extension.

## Methods

In this section, we describe our proposed algorithm that learns the embeddings of medical terms. We first define the problem and briefly introduce the baseline skip-gram algorithm [16], which is the basis of our approach. Then, we describe our proposed algorithm.

### Problem definition

Let us suppose we are given a corpus of medical notes. We describe a single note *N* as an ordered sequence of terms,* N* = {*w*_*1*_,* w*_*2*_,* …*,* w*_*n*_}, where *w*_*i*_ is a term from vocabulary *V* and *n* is the length of the note. The size of the vocabulary is |*V*|. A term can be a single word (e.g., *Parkinson*) or a multi-word (e.g., *Parkinson’s disease*). The objective of term embedding is to represent each term from the vocabulary as a vector, such that semantically similar terms have similar vectors.

### Skip-gram algorithm

**Fig. 1:**
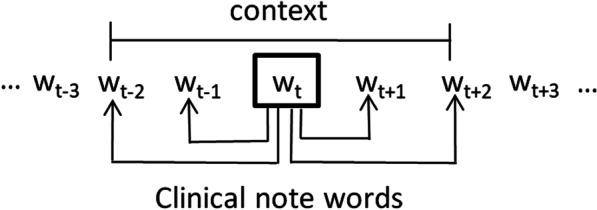
The framework for the skip-gram algorithm

The skip-gram algorithm for embedding [16] scans the terms in a note and updates their vector representations based on their context. The context of a term is typically defined as its neighboring terms in a sequence. Given the target term *w*_*t*_ from the corpus, the skip-gram algorithm creates term pairs consisting of the scanned term *w*_*t*_ and its context terms *w*_*i*_, and uses pairs (*w*_*t*_,* w*_*i*_) to update the likelihood of observing the context term *w*_*i*_ given the target term *w*_*t*_. The context of *w*_*t*_ is defined as its neighboring terms *C*_*wt*_ = (*w*_*t−2*_,* w*_*t−1*_,* w*_*t*+*1*_,* w*_*t*+*2*_), if the context size is 2. Context terms *w*_*i*_ are selected from *C*_*wt*_. The log-likelihood of observing context terms for all the terms in the corpus is defined as

1 $$L = \mathop \sum \limits_{{t, w_{i} \in C_{w_{t}} }}^{{}} \log p{(}w_{i} {|}w_{t} )$$

where *p*(*w*_*i*_*|w*_*t*_) is the conditional probability of context term *w*_*i*_ given the target term *w*_*t*_. The skip-gram approach is illustrated in Fig. 1.

In order to model *p*(*w*_*i*_*|w*_*t*_), skip-gram assigns vectors *U*_*w*_ and *V*_*w*_ to term *w* from the vocabulary. The dimension of both vectors is the same. The conditional probability is defined as the following softmax function

2 $$P{(}w_{i} {|}w_{t} ) = \frac{{e^{{U_{{w_{t} }} . V_{{w_{i} }} }} }}{{\mathop \sum \nolimits_{{ w_{j} \in \left| V \right| }}^{{}} e^{{U_{{w_{t} }} . V_{{w_{j} }} }} }}$$

where the dot product between two vectors is used to measure the similarity between two terms. A gradient descent algorithm can be used to maximize the objective function of Eq. (1). However, since the computational complexity of calculating Eq. (2) is very high due to its denominator, skip-gram uses negative sampling where the log-likelihood objective function is replaced with the negative sampling instantaneous loss for each target word *w*_*t*_, defined as

3 $$E_{t} = \mathop \sum \limits_{{i \in C_{w_{t}} }} \left( { - \log \sigma \left( {U_{{w_{t} }} \cdot V_{{w_{i} }} } \right) - \mathop \sum \limits_{{w_{j} \in W_{neg} }} - \log \sigma \left( {U_{{w_{t} }} \cdot V_{{w_{j} }} } \right)} \right)$$

where

4 $$\sigma \left( {U_{{w_{t} }} \cdot V_{{w_{x} }} } \right) = \frac{1}{{1 + e^{{ - U_{{w_{t} }} \cdot V_{{w_{x} }} }} }}.$$

Here, *W*_*neg*_ is a set of *K* so-called negative terms randomly sampled from the corpus. Skip-gram uses a stochastic gradient algorithm to greedily maximize the instantaneous loss. After the training is finished, vector *U*_*w*_ is used as an embedding for term *w*.

### Our proposed method: *definition2vec*

The proposed *definition2vec* algorithm enhances the skip-gram approach by exploiting the textual definitions of medical terms available in public resources. Similar to skip-gram, it scans the terms in a corpus and uses stochastic gradient descent to minimize the negative sampling instantaneous loss. However, when updating the embedding of a term, *definition2vec* also accounts for embeddings from its definition.

Let us assume target term *w*_*t*_ has its definition in a form of a word sequence *D*_*wt*_ = (*d*_*1*_,* d*_*2*_,* …*,* d*_*m*_), where *d*_*i*_ is the *i*-th definition word of *w*_*t*_ and *m* is the length of the definition. We denote *z*_*d*_ as the vector representation of word d from the definition and *U*^*′*^_*wt*_ as the definition-independent vector for the target term. We express the resulting target vector as

5 $$U_{{w_{t} }} = \frac{{sqrt\left( {f_{{w_{t} }} } \right)U_{{w_{t} }}^{{\prime }} + \beta \frac{{\mathop \sum \nolimits_{{d \in D_{{w_{t} }} }} z_{d} }}{{\left| {D_{{w_{t} }} } \right|}}}}{{sqrt\left( {f_{{w_{t} }} } \right) + \beta }}$$

**Fig. 2:**
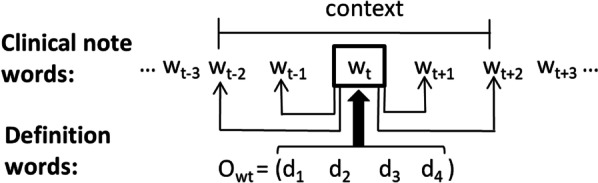
Architecture of the proposed *definition2vec* algorithm

Here, *f*_*wt*_ is the frequency of *w*_*t*_ in the corpus and *β* is a hyperparameter. By using Eq. 5, our goal is to obtain the embedding of *w*_*t*_ that is influenced by its context and definition. Figure 2 illustrates the proposed approach. If a term frequently occurs in the corpus, its representation will be influenced more strongly by its contextual terms than its definition words. However, if a term is rare or unseen in the corpus, its representation will be heavily influenced by its definition words. Hyperparameter *β* determines the impact of a term’s definition on its embeddings. Our proposed algorithm scans the corpus term by term and constructs pairs of context and target terms together with their corresponding negative pairs. It follows the negative sampling idea of skip-gram and uses a stochastic gradient algorithm to minimize the instantaneous loss. The updates of context term, target term, and definition word vectors are calculated as follows,

6 $$V_{{w_{x} }} = V_{{w_{x} }} - \alpha \frac{dE}{{d\left( {V_{{w_{x} }} } \right)}}$$

7 $$\frac{dE}{{d\left( {V_{{w_{x} }} } \right)}} = \frac{dE}{{d\left( {U_{{w_{t} }} V_{{w_{x} }} } \right)}}\frac{{d\left( {U_{{w_{t} }} V_{{w_{x} }} } \right)}}{{d\left( {V_{{w_{x} }} } \right)}}$$

8 $$U_{{w_{t} }}^{{\prime }} = U_{{w_{t} }}^{{\prime }} - \alpha \frac{dE}{{d\left( {U_{{w_{t} }}^{{\prime }} } \right)}}$$

9 $$\frac{dE}{{d\left( {U_{{w_{t} }}^{\prime } } \right)}} = \mathop \sum \limits_{{w_{x} \in \left( {w_{i} \cup W_{neg} } \right)}} \frac{dE}{{d\left( {U_{{w_{t} }} V_{{w_{x} }} } \right)}}\frac{{d\left( {U_{{w_{t} }} V_{{w_{x} }} } \right)}}{{d\left( {U_{{w_{t} }} } \right)}}\frac{{d\left( {U_{{w_{t} }} } \right)}}{{d\left( {U_{{w_{t} }}^{\prime } } \right)}}$$

10 $$z_{d} = z_{d} - \alpha \frac{dE}{{d\left( {z_{d} } \right)}}$$

11 $$\frac{dE}{{d\left( {z_{d} } \right)}} = \mathop \sum \limits_{{w_{x} \in \left( {w_{i} \cup W_{neg} } \right)}} \frac{dE}{{d\left( {U_{{w_{t} }} V_{{w_{x} }} } \right)}}\frac{{d\left( {U_{{w_{t} }} V_{{w_{x} }} } \right)}}{{d\left( {U_{{w_{t} }} } \right)}}\frac{{d\left( {U_{{w_{t} }} } \right)}}{{d\left( {z_{d} } \right)}}$$

where *α* is the learning rate.

After the training is finished, the target vector *U*_*w*_ becomes an embedding for term *w*. As a by-product of the learning procedure, we also learn the embeddings of each definition word.

## Results

In this section, we start by explaining the data sets and data preprocessing. Then, we describe the experimental design. Finally, we show and discuss the results of our qualitative and quantitative evaluation.

### Data sets

In our experiments, we used two data sets. The first data set is the UMLS Metathesaurus, which has textual definitions for a large number of medical terms. The second data set is MIMIC-III, which contains EHR records of a large number of Intensive Care Unit (ICU) patients with notes written in English.

UMLS Metathesaurus: Unified Medical Language System (UMLS) is a set of files and software that integrates multiple medical vocabularies [11]. UMLS Metathesaurus is the component of UMLS that maintains medical concepts and their textual definitions which are linked to different medical source vocabularies such as National Cancer Institute Thesaurus (NCIT) [36], Medical Subject Heading (MeSH) [35], Universal Medical Device Nomenclature System (UMD) [37], Human Phenotype Ontology (HPO) [38] and Mondo Disease Ontology (MONDO) [39]. UMLS Metathesaurus lists 188,050 concepts with at least one textual definition, each with its Concept Unique Identifier (CUI). Each concept has one or more medical terms associated with it, where each term has its String Unique Identifier (SUI). Each SUI can have one or more Atomic Unique Identifiers (AUI) that link the term to its definition from a particular source vocabulary. UMLS Metathesaurus has 773,692 SUIs. Although there are over 2.5 million medical concepts listed in UMLS Metathesaurus, in this study, we only consider those with at least one definition because *definition2vec* requires them.

MIMIC-III: Medical Information Mart for Intensive Care (MIMIC-III) is a publicly available deidentified data set that contains EHRs of 41,127 ICU patients from Beth Israel Deaconess Medical Center recorded between 2001 and 2012 [40]. This data set contains both structured (medical codes, lab results) and unstructured (medical notes) data. MIMIC-III contains several types of medical notes such as progress notes, radiology reports, and discharge summaries. In this study, we only consider discharge summaries prepared by a health provider at the conclusion of an ICU stay. There is a total of 59,652 discharge summaries in MIMIC-III indicating that most patients have a single EHR in the data set. In our study, we are also interested in ICD-9-CM diagnosis codes [41] listed with each patient stay in the MIMIC-III data set. There is a total of 6,717 unique diagnosis codes listed in the data set.

### Data processing

**Fig. 3:**
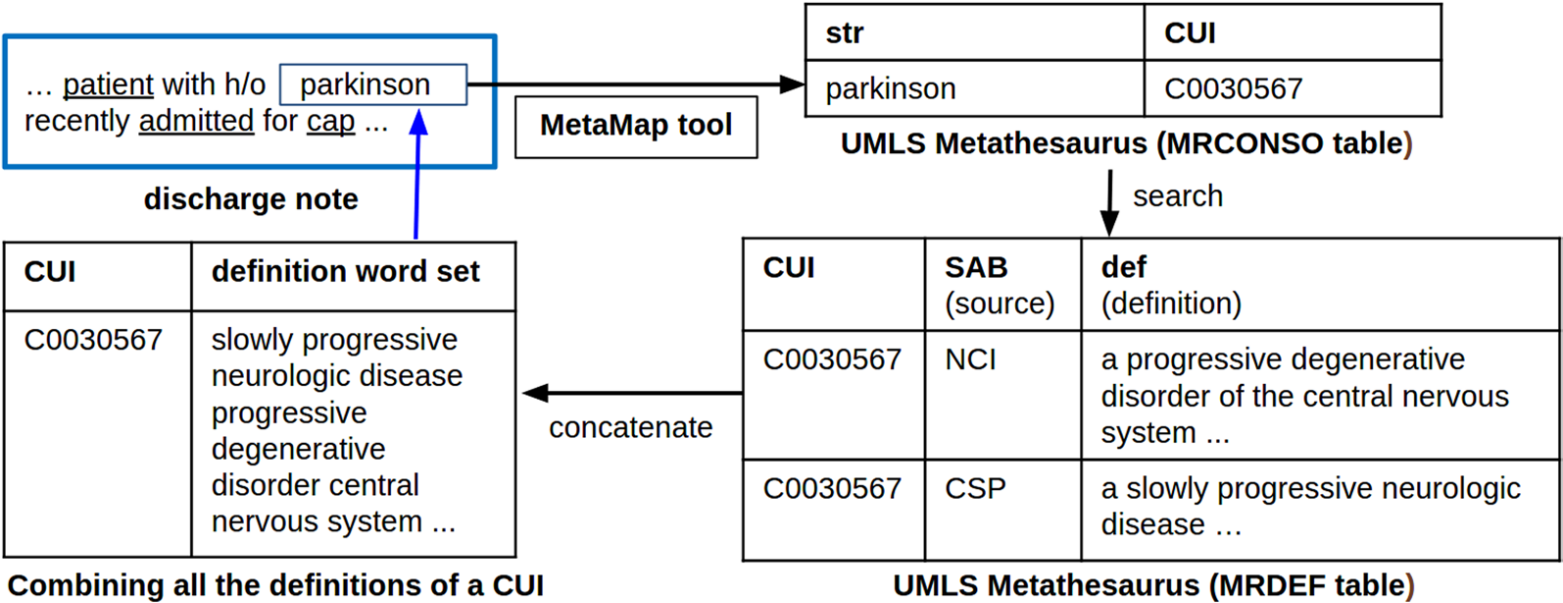
Illustrating a process for extracting definitions of medical terms

Given a discharge summary, we performed several preprocessing steps illustrated in Fig. 3. First, we removed digits and special characters, converted all characters into lower case, and tokenized the text. Then, we used MetaMap v16.2 [42] to automatically match the tokens with UMLS CUIs. Each token can remain unmatched, become directly matched to a medical concept, or become a part of a multi-word phrase that is matched to a medical concept. If a matched concept is a multi-token such as “*Parkinson disease*” we concatenated the tokens into a single token by adding an underscore special character such as “*Parkinson disease*”. Finally, we removed all unmatched tokens, such that each discharge summary becomes a sequence of tokens matched with medical concepts from UMLS Metathesaurus. This preprocessing procedure matches the previous work [12].

To find definitions of each matched token, we performed the following steps. First, we identified the CUI of each matched token. Then, we found all AUIs corresponding to the CUI, retrieved the medical term definition of each AUI, and concatenated the definitions. Finally, we preprocessed the definition sentences to remove digits and special characters, lowercase all characters, tokenize, and remove stop words and rare words. Figure 3 illustrates the process that starts from a discharge note and ends with a sequence of CUI-matched tokens with their corresponding definitions.

### Learning medical term embeddings

After preprocessing the discharge summaries from MIMIC-III following the procedure illustrated in Fig. 3, each medical term in the resulting corpus is linked to its definition sequence. In this subsection, we describe experimental design that was used to produce embeddings by *definition2vec* and the baseline algorithms.

Our first step was to split the set of preprocessed discharge summaries randomly into training, validation, and test sets. Similar to [7], the resulting training data set contained 47,423 notes from 36,998 patients, test data had 3372 notes from 2,755 patients, and validation set had 1632 notes from 1374 patients. One patient can have their discharge notes in only one of the three subsets.

We used the training data set to learn the embeddings of medical terms. In this way, we learned the embeddings of 46,861 medical terms corresponding to 29,740 medical concepts. Some statistics about the training data set are listed in Table 1. We trained *definition2vec* and the baselines on the preprocessed training data to learn medical term embeddings. We used Python Gensim implementation of three popular embedding algorithms as baselines: GloVe,^1^ skip-gram,^2^ and fastText.^3^

**Table 1:** Statistics of discharge summaries in the MIMIC-III training data

# training notes	47,423
# of unique medical terms in training data	46,861
Average # of medical terms in a discharge summary	671
# of unique medical concepts in training data	29,740
Average # of medical concepts per discharge summary	364
Average # of definition words per medical concept	16
# of unique diagnosis codes in training data	6717
Average # of diagnosis codes per discharge summary	11

We used the same hyperparameters for all embedding algorithms: word context neighborhood (or window size) = 5, embedding vector length (or feature size) = 100, learning rate = 0.01, number of negative samples = 5. Those same parameters had been used in previous research [16, 43]. All models were trained for 10 epochs, which was sufficient for convergence.

Glove, skip-gram, fastText, and *definition2vec* embeddings are non-contextualized, meaning that every term has a fixed vector representation. In contrast, recent research resulted in contextualized embeddings, where vector representation of a given term depends on the context in which it is mentioned. The most notable representative of contextualized embeddings is BERT neural network [44], which was trained on a large corpus of general-purpose text. In particular, given an input text, the final hidden layer of BERT provides a 768-dimensional embedding for every WordPiece [45] token. Each word can be represented with the embedding of the first WordPiece token of the word. Such embedding is contextualized. A recent study [46] found that the BERT contextualized embeddings can outperform context-free embeddings from skip-gram, fastText, and GloVe in several downstream tasks. Thus, we compared BERT embeddings with the non-contextualized embeddings in our experiments.

To get BERT contextual-embedding of medical terms in a discharge note, we sent the lowercased note to the BERT model and recorded the embedding of every medical term. If the discharge note has more than 512 tokens, we first divided it into subsequences shorter than 512 and concatenated medical term embeddings from all the subsequences.

We performed two types of studies to evaluate the baseline and our proposed term embeddings, as explained next.

### Downstream evaluation: predicting ICD-9-CM diagnosis codes

Our first evaluation was to use the embeddings in a downstream task of predicting ICD-9-CM diagnostic codes for a given discharge summary. This is a multi-label classification where the prediction model provides multiple outputs, one for each ICD-9-CM diagnosis code. The employed prediction model was Convolutional Attention for Multi-Label classification (CAML) [7], which is a convolutional neural network (CNN) with the attention mechanism. In CAML, each medical term from the pre-processed discharge summary is converted to a vector according to its embedding and provided as an input to the neural network. The output of CAML is a binary vector of predictions of ICD-9-CM diagnosis codes.

For measuring the accuracy, we use “recall at 8”, micro-averaged (MIC) and macro-averaged (MAC) F1, and area under the ROC curve (AUC), similar to the previous research [7]. Recall at k (k = 8), is the fraction of correctly predicted ICD-9-CM diagnosis codes among the k most confidently predicted codes. To calculate F1, we must first calculate recall and precision. Recall is a fraction of true ICD-9-CM diagnosis codes predicted by CAML. Precision is a fraction of true ICD-9-CM diagnosis codes among the predicted codes. The F1 score is measured by the harmonic mean of recall and precision. In MIC calculations, each pair (discharge note, code) is taken as a separate prediction. Then, all predictions are used to calculate the F1 accuracy. On the other hand, the MAC values are computed by first calculating F1 on each individual ICD-9-CM diagnosis code. Then, the code- specific F1 accuracies are averaged to obtain the MAC F1 accuracy [7]. Compared to MIC F1 accuracy, the MAC F1 accuracy places a higher emphasis on rare code predictions.

We used the CAML implementation provided by the authors.^4^ We used the learned embeddings of our proposed method and the three non-contextualized baselines as input for CAML. The embeddings were not modified during CAML training. All trained models had identical neural network architecture and the default hyperparameters given in the original paper. Each CAML was trained on all available training data. We checked the “recall at 8”, accuracy on the validation set after each epoch as stopping criteria. If the “recall at 8” value did not increase after ten consecutive epochs, we stopped the training. For *definition2vec*, we tuned *β* value by exploring different values i.e., 1, 2, 5, 10, 20, 50, and 100. Based on the validation data, we obtained the best results for *β* = 10. The CAML model had 2690 outputs with sigmoid neurons, corresponding to all ICD-9-CM diagnosis codes with frequency ≥ 10 in our training data.

To evaluate the contextualized BERT embeddings, we also used the same CAML architecture and training procedures. The only difference was the dimensionality of the embeddings, which was 768 for BERT versus 100 for the non-contextualized embeddings.

The results in Table 2 show accuracy measured on test data. It can be observed that *definition2vec* is more accurate than the baselines on the F1 MAC measure, while it is comparable to skip-gram and fastText on other accuracy measures. We note that the F1 MAC accuracy gives a larger weight to rare ICD-9-CM diagnosis codes than the F1 MIC measure.

**Table 2:** Accuracy of ICD-9-CM diagnosis code prediction using large training data set (predicting top 2690 ICD-9-CM diagnosis codes having frequency at least 10 times in training data)

Model	AUC		F1		
	MIC	MAC	MIC	MAC	R@8
BERT	0.9580	0.8769	0.4516	0.0932	0.3922
GloVe	0.9703	0.8888	0.4727	0.1126	0.3938
skip-gram	0.9790	0.9316	0.4995	0.1333	0.4147
fastText	**0.9794**	0.9340	0.4950	0.1372	0.4168
definition2vec	**0.9794**	**0.9350**	**0.5065**	**0.1489**	**0.4173**

Bold font emphasizes the best method for each accuracy category

The results also show that BERT contextualized embeddings are not better than the non-contextualized *definition2vec* embeddings. We think that the main reason is that BERT was pre-trained on large general-purpose corpus while the *definition2vec* and the other baseline methods (i.e., GloVe, skip-gram, and fastText) were trained on a specialized discharge note corpus.

### Downstream evaluation: predicting ICD-9-CM diagnosis codes using small training data

In many medical informatics applications, the available corpus is much smaller than the MIMIC-III data set. Our hypothesis is that *definition2vec* is very appropriate for small data scenarios where most of medical terms are not observed often enough to enable baseline algorithms to learn good embeddings.

We repeated the CAML experiments described in the previous subsection using smaller training data sets. In particular, we created four training data sets by randomly sampling 1000, 2000, 5000, and 10,000 discharge summaries from the training data. We trained *definition2vec* and the baselines (GloVe, skip-gram, fast- Text) on the small data sets for 40 iterations to learn concept embedding with the same parameters as before (window size = 5, feature size = 100, learning rate = 0.01, and number of negative samples = 5).

After learning the representations of medical terms, we trained a CAML model in the same manner, using the full training data set. We only predicted ICD-9-CM diagnosis codes that occurred at least 10 times in the training data set. For each size of training data, we used validation to determine the best choice for *β* in *definition2vec* from among the following choices.

*β* = 1, 2, 5, 10, 20, 50, and 100. We found *β* = 50 gives the best results for 1000 and 2000 data sets, *β* = 20 is the best choice for the 5000 data set, and *β* = 10 for the 10,000 data set.

Table 3 shows CAML accuracy for each data set. For all four small training data sets, *definition2vec* outperforms the baselines on all metrics. The difference between *definition2vec* and the baseline methods is particularly large on the two smallest training data sets (1000 and 2000) and the difference reduces on the two largest training data sets (5000 and 10,000). Therefore, Table 3 results strongly support our hypothesis that *definition2vec* is particularly useful on small corpora.

**Table 3:** Accuracy of ICD-9-CM diagnosis code prediction using small training data sets (UT: number of unique medical terms, DC: number of ICD-9-CM diagnosis codes, PDC: number of predicted ICD-9-CM diagnosis codes occurring at least 10 times in training data)

Model	1000 data set UT: 9632 DC: 1351 PDC: 138					5000 data set UT: 19,601 DC: 3114 PDC: 500				
	AUC		F1			AUC		F1		
	MIC	MAC	MIC	MAC	R@8	MIC	MAC	MIC	MAC	R@8
GloVe	0.8240	0.6919	0.1546	0.0266	0.3560	0.9122	0.8386	0.2829	0.0805	0.3997
BERT	0.8368	0.7212	0.1675	0.0341	0.3588	0.9198	0.8389	0.3016	0.1013	0.4063
skip-gram	0.8409	0.7426	0.1440	0.0320	0.3797	0.9439	0.9002	0.4274	0.2056	0.4621
fastText	0.8414	0.7720	0.1968	0.0711	0.4001	0.9468	0.9053	0.4291	0.2081	0.4663
definition2vec	**0.8587**	**0.7958**	**0.2583**	**0.0985**	**0.4323**	**0.9475**	**0.9066**	**0.4314**	**0.2108**	**0.4696**

	2000 data set UT: 13,551 DC: 1932 PDC: 272					10,000 data set UT: 26,738 DC: 4186 PDC: 1100				
	AUC		F1			AUC		F1		
	MIC	MAC	MIC	MAC	R@8	MIC	MAC	MIC	MAC	R@8
GloVe	0.8505	0.7512	0.2175	0.0500	0.3306	0.9496	0.8761	0.4257	0.1355	0.4352
BERT	0.8636	0.7731	0.2022	0.0466	0.3431	0.9427	0.8743	0.3680	0.0970	0.3827
skip-gram	0.8709	0.7873	0.2050	0.0312	0.3455	0.9604	0.9105	0.4539	0.1796	0.4445
fastText	0.8722	0.7929	0.2059	0.0362	0.3539	**0.9613**	0.9128	0.4554	0.1847	0.4472
definition2vec	**0.8891**	**0.8338**	**0.2915**	**0.1055**	**0.3985**	**0.9613**	**0.9136**	**0.4564**	**0.1875**	**0.4488**

Bold font emphasizes the best method for each accuracy category

In addition, we found that larger *β* in *definition2vec* were appropriate for smaller training data sets and vice versa. This result supports our hypothesis that if a term is rare or unseen in the training corpus, its representation should be heavily influenced by its definition words.

### Semantic similarity evaluation: 3 human labeled data sets

Several studies [12, 27] used similarity scores between pairs of medical concepts or terms to evaluate learned embeddings. For the evaluation of our learned embeddings, we used three different data sets as described below.

Pedersen data set: Pedersen [47] provides a data set of 30 UMLS medical term pairs with semantic similarity judgments by 3 physicians and 9 clinical terminologists.

Pakhomov data set: This data set [48] consists of 101 clinical term pairs whose similarity was determined by 9 medical coders and 3 physicians from Mayo Clinic.

UMNSRS data set: The UMNSRS data set [49] has 566 medical term pairs. Each medical term pair has a semantic similarity score determined by 8 medical residents from the University of Minnesota Medical School.

For this experiment, we treated all strings in the three data sets as medical terms and we matched them with our embeddings. To compare the embeddings, we measured the cosine similarity between them and calculated the Pearson correlation coefficient between the cosine similarity scores and the scores by the human experts. Some medical terms from the three data sets do not exist in the vocabulary of our learned embeddings. Thus, we used 25, 67, and 306 medical term pairs from the three semantic similarity data sets, respectively. Since BERT is a contextual embedding model that provides different vectors for the same term in different contexts, we did not include this model as a baseline for this experiment. Table 4 shows the Pearson correlation coefficients for *definition2vec* and baseline methods. The results indicate that *definition2vec* better reflects the underlying semantic relationships between the medical terms.

**Table 4:** Pearson correlation coefficient for semantic pair similarity

Data set	GloVe	skip-gram	fastText	definition2vec
Pedersen	0.2963	0.4297	0.6256	**0.6468**
Pakhomov	0.1712	0.5310	0.5732	**0.5888**
UMNSRS	0.2182	0.6058	0.6188	**0.6392**

Bold font emphasizes the best method for each accuracy category

### Semantic similarity evaluation: UMLS semantic types

UMLS semantic network has 127 different semantic types such as “*drug*”, “*virus*”, “*disease*”, and “*procedure*”, which categorize medical concepts and reveal the relationships between them. We labeled each of the embedded medical terms into one of the 127 classes. Then, we applied a *k*-means clustering algorithm with *k* = 127 on the embeddings learned from the full training data set. We used normalized mutual information (NMI) to evaluate the purity of the clusters with respect to their semantic network labels. A high NMI value indicates that the clusters are pure and contain a limited set of semantic types in each cluster.

Table 5 compares the NMI values obtained with four different embedding algorithms. Clusters obtained with GloVe embeddings have the lowest conformity with semantic labels. Clusters obtained with *definition2vec* embeddings show the largest conformity. The clusters obtained with fastText were similar to *definition2vec’s*, with slightly less conformity. The results indicate that *definition2vec* is successful in keeping similar medical terms close together in the learned vector space.

**Table 5:** Cluster NMI value for different models

Model	NMI value
GloVe	0.1339
Skip-gram	0.2130
fastText	0.2834
definition2vec	**0.3054**

Bold font emphasizes the best method for each accuracy category

### Qualitative evaluation

We learned *definition2vec* and baseline embeddings on the full training data set (47,423 summaries) and on the smallest training data set (1,000 summaries). Then, we searched the nearest neighbors in the embedding space for a range of medical terms. For a given medical term, we found its 10 nearest neighbors based on the cosine similarity between the embeddings. For example, Table 6 shows the nearest neighbor terms of “*heart attack*” based on learning from the full and the smallest training data sets. For the full data set, both *definition2vec* and skip-gram provide similar results, with *blockage*, *heart muscle*, *heartblockage*, and slow *heart rate* in the results of both methods. However, the results based on the smallest training data set are different. *definition2vec* finds *myocardial infarctions*, *acute mi*, *hemorrhagic stroke*, and *hypertensive crisis*, which are all the concepts related to “*heart attack*”. On the other hand, skip-gram finds pain, cough, blood, scheduling, skip, and cell phone, which are not as closely related to “heart attack”.

**Table 6:** Showing top 10 nearest neighbor terms for “*heart attack*” in *definition2vec* and skip-gram

Large data set		Small data set	
definition2vec	skip-gram	definition2vec	skip-gram
blockage	blocked artery	myocardial infarctions	pain
heart muscle	blockage	acute mi	cough blood
heart attacks	heart blockage	infarction	scheduling
heart blockage	heart muscle	hemorrhagic stroke	aortic aneurysms
blocked heart	blocked heart	myocarditis	abuse substance
heart block diagnosis	heart muscles	hypertensive crisis	providers
block heart	blood clots lung	myocardial	skip
slow heart rate	heart function	restrictive cardiomyopathy	caregiver
heart function	slow heart rate	ischemic change	substance abuse problem
myocardia	myocardial infarction	ischemia	cell phone

Table 7 shows another example with the nearest neighbors of “*bipolar disorder*”. Similar to the previous example, *definition2vec* and baseline embeddings result in similar neighborhoods when trained on the full training data set. For example, the top neighbors for both methods are *schizophrenia*, *schizoaffetive disorder*, *bpad*, and *mood disorder*. However, the results obtained by learning on the smallest training data set are different. *definition2vec* finds several concepts that are related to the “*bipolar disorder*”, such as *depression*, *psychosis*, and *hyperlipidemia*, while the nearest neighbors found by skip-gram are less related, such as *armour*, *parkinson disease*, and *ckd* (abbreviation of *chronic kidney disease*). From these results, we can conclude that *definition2vec* provides similar embeddings to skip-gram when both are trained on the full training data set, while it seems to be superior when the training data set is small.

**Table 7:** Showing top 10 nearest neighbor terms for “*bipolar disorder*” in *definition2vec* and skip-gram

Large data set		Small data set	
definition2vec	skip-gram	definition2vec	skip-gram
schizophrenia	schizophrenia	depression	armour
schizoaffective disorder	schizoaffective disorder	psychosis	parkinson disease
major depression	depression	asthma	sildenafil
paranoid schizophrenia	major depression	hyperlipidemia	addison disease
bpad	bpad	neuropathy	ckd
psychotic disorder	multiple personality disorder	diabetic neuropathy	amenorrhea
bipolar affective disorder	seizure disorder	dyslipidemia	renal carcinoma
mood disorder	mood disorder	hypertension	obesity hypoventilation syndrome
bipolar illness	pervasive developmental disorder	malignant hypertension	oa
bipolar mood disorder	paranoid schizophrenia	anxiety	esophageal dilatation

### Qualitative evaluation: out-of-vocabulary (OOV) medical terms

There might be many important medical terms that do not occur in the training data, but have definitions in UMLS Metathesaurus. Since *definition2vec* learns word embeddings through medical term definitions, it can calculate the embeddings of OOV terms by taking the average of their definition word embeddings. For example, in Table 8 we show the top 10 neighbors of “*nicotine replacement therapy*” and “*gastric pains*” which do not occur in the full training data set. *definition2vec* properly finds “*nicotine replacement*”, “*smoking cessation therapy*”, “*nicotine patches*” among the nearest neighbors of the OOV “*nicotine replacement therapy*” term. Similarly, it properly identifies neighbors of the OOV term “*gastric pains*.” These results show that *definition2vec* can find the proper embeddings of OOV medical terms using definition word embeddings. This puts *definition2vec* at an advantage over Glove and skip-gram, which cannot provide embeddings for OOV terms. It also has an advantage over fastText, which relies purely on n-gram embeddings to calculate the embeddings of OOV terms.

**Table 8:** Showing top 10 nearest neighbor terms for two OOV terms, “*nicotine replacement therapy*” and “*gastric pains*” in *definition2vec*

nicotine replacement therapy	gastric pains
nicotine replacement	stomach ache
smoking cessation therapy	stomach pain
nicotine patches	feeling bloated
nicotine transdermal patch	pain esophagus
ceassation smoking	gastrointestinal pain
nicotine dependence	esophageal pains
nicotine addiction	abdominal pains
quiting smoking	low ache
nicotine lozenges	low pains
dependence nicotine	gi pain

## Discussion

Often in practice, a document corpus is too small for training language models and is only useful for learning embeddings of the most common terms. To address this issue, we extended the skip-gram algorithm to incorporate the definitions of medical terms from external publicly available resources. In our case, we relied on the UMLS Metathesaurus as the external source. We note that the proposed *definition2vec* algorithm allows other sources of medical term definitions, including web resources such as Wikipedia.

Our experiments show that *definition2vec* results in better medical term embeddings, especially when the size of a document corpus is small. This could be particularly useful in applications [19–21] where it is not feasible to have a large corpus, such as when the corpus is from a specialized medical practice, is related to the treatment of a rare medical condition, or is written in a rare language. *Definition2vec* could also be applicable to non-medical domains such as the embeddings of legal terms or specialized terms used in various scientific domains.

Recent advances in contextualized embedding represented by neural networks such as ELMo (Embedding from Language Models) [50] and BERT (Bidirectional Encoder Representations from Transformers) [44] allow embeddings to depend on the context of each term’s occurrence. Although a recent study [46] found that the BERT contextualized embeddings can be superior to context-free embeddings from skip-gram, fastText, and GloVe in some applications, our results indicate that in a small and specialized corpus setting it does not have to be the case. Another recent paper [51] also reported that BERT embeddings did not improve prediction accuracy on a medical code prediction task. We believe that this is because BERT is trained on general-purpose corpus that does not provide sufficient information to capture useful representations of highly specialized medical terms.

### Limitations

The proposed study has some limitations. For example, there are versions of BERT specialized for medical text, such as ClinicalBERT [52], which was fine-tuned on all MIMIC-III medical notes. However, ClinicalBERT was not appropriate for our experiments, because we wanted to compare embeddings that could be learned on very small subsets of MIMIC-III. Thus, we had to constrain our evaluation to BERT contextualized embeddings.

Moreover, the presented experiments relied on MetaMap to match the text with medical concepts. MetaMap does not provide perfect coverage of medical terms, most often due to spelling mistakes or non-standard jargon or abbreviations. To enable matching of non-standard term variants, it might be helpful to consider character-level embedding neural networks trained to reconstruct, or mimic, an embedding from a word-level embedding model [53].

## Conclusions

In this paper, we proposed a new algorithm, *definition2vec*, which learns medical term embeddings by combining a data set of discharge summaries and definitions of medical terms. We evaluated the learned embeddings by comparing their usefulness when predicting medical codes from discharge summaries and how closely they match semantic similarities between medical terms. Our results indicate that *definition2vec* is particularly useful in downstream task when the training data set is small. Moreover, the medical term definitions are especially beneficial for the embedding of rarely seen or out-of-vocabulary medical terms. Hence, the proposed method can be useful for analysis of rare medical conditions and treatments from EHR data.

## Data Availability

We used two data sets in our experiment. The first data set is the UMLS Metathesaurus that can be found in (https://www.nlm.nih.gov/research/umls/licensedcontent/umlsknowledgesources.html). The second data set (the MIMIC-III data set) can be obtained in (https://mimic.physionet.org/). Researchers seeking to use the databases must formally request access following the steps on their websites.

## Abbreviations

BERT: Bidirectional Encoder Representations from Transformers
CBOW: Continuous Bag of Words
CAML: Convolutional Attention for Multi-Label classification
EHR: Electronic Health Record
HPO: Human Phenotype Ontology
ICD: International Classification of Diseases
ICD-9-CM: International Classification of Diseases, Ninth Revision, Clinical Modification
MeSH: Medical Subject Heading
MIMIC: Medical Information Mart for Intensive Care
MONDO: Mondo Disease Ontology
NCIT: National Cancer Institute Thesaurus
NLP: Natural Language Processing
NN: Neural Network
NMI: Normalized Mutual Information
UMLS: Unified Medical Language System
UMD: Universal Medical Device Nomenclature System

## Footnotes

1 https://radimrehurek.com/gensim/scripts/glove2word2vec.html.

2 https://radimrehurek.com/gensim/models/word2vec.html.

3 https://radimrehurek.com/gensim/models/fasttext.html.

4 https://github.com/jamesmullenbach/caml-mimic
